# Boost Your Brain Health: Methods and Baseline Characteristics for a Randomized, Controlled Trial of an Online Study to Reduce Dementia Risk Factors

**DOI:** 10.1002/alz.092609

**Published:** 2025-01-09

**Authors:** Patricia Turo, Cynthia Benjamin, Henry Brodaty, Rebecca L Sudore, Margaret A Chesney, Linda L Chao, Deborah E Barnes

**Affiliations:** ^1^ Together Senior Health, New York, NY USA; ^2^ Centre for Healthy Brain Ageing (CHeBA), University of New South Wales, UNSW Sydney, NSW Australia; ^3^ University of California, San Francisco, San Francisco, CA USA; ^4^ San Francisco Veterans Affairs Medical Center, San Francisco, CA USA

## Abstract

**Background:**

Nearly 1 in 3 older adults are living with mild cognitive impairment (MCI) or subjective cognitive decline (SCD), which places them at increased risk of developing Alzheimer’s disease or related dementias (ADRD). Behavioral interventions can improve cognitive function and reduce dementia risk factors, but most are in‐person and time‐intensive. We are conducting a randomized, controlled trial to assess the impact of 12‐ and 24‐week online interventions on cognitive function and dementia risk.

**Method:**

Study participants are English‐speaking adults aged 55 years or older living in the U.S. who self‐report MCI or SCD, have 2 or more modifiable ADRD risk factors, and have an electronic device with video camera to participate in online classes. Participants are randomized to Brain Health Academy (asynchronous expert videos, 30 hours over 12 weeks), Brain Health Together (synchronous group Brain Health Education and Moving Together classes + individual brain health coaching, 30 hours over 12 weeks), or Brain Health Together Plus (Brain Health Together plus an additional 12 weeks of group classes and individual brain health coaching, 45 hours over 24 weeks). We used descriptive statistics to summarize self‐reported demographics and motivators and barriers to participation.

**Result:**

Of 397 individuals who have completed screening, 40% were ineligible, 25% declined, and 35% enrolled and have been randomized to date. Enrolled participants have a mean (SD) age of 70 (8) years and 3 (1) modifiable risk factors; 75% are female, 24% are non‐White or Hispanic, and 23 states from across the US are represented. The greatest motivators to study participation are wanting to keep one’s brain as healthy as possible (89%) and being worried about changes to memory or thinking (81%). Time commitment was perceived as the greatest barrier (30%). Most participants indicated they were already making changes to improve brain health.

**Conclusion:**

Many older adults with MCI/SCD and modifiable risk factors for ADRD are interested in participating in online brain health programs and are highly motivated to make lifestyle changes to keep their brains healthy. We anticipate that enrollment will be completed by May 2024.

